# High Variability of the Definition of Recurrent Glenohumeral Instability: An Analysis of the Current Literature by a Systematic Review

**DOI:** 10.1016/j.asmr.2021.02.002

**Published:** 2021-04-06

**Authors:** Hassanin Alkaduhimi, James W. Connelly, Derek F.P. van Deurzen, Denise Eygendaal, Michel P.J. van den Bekerom

**Affiliations:** a Shoulder and Elbow Unit, Joint Research, Amsterdam, the Netherlands; b Department of Orthopaedic Surgery, Massachusetts General Hospital, Boston, Massachusetts, U.S.A.; c Orthopaedic Department, Amphia Ziekenhuis, Breda, the Netherlands; d Amsterdam University Medical Centres, Amsterdam, the Netherlands

## Abstract

**Purpose:**

To determine the definitions for recurrence used in the literature, assess the consensus in using these definitions, and determine the impact of these definitions on recurrence rates.

**Methods:**

A literature search was performed in PubMed and EMBASE including studies from 2000 to 2020 reporting on recurrence rates after anterior arthroscopic shoulder instability surgery. Dislocation, apprehension, subluxation and recurrence rates were compared.

**Results:**

Ninety-one studies were included. In 68% of the eligible studies, recurrence rates are not well defined. Thirty (33%) studies did not report on dislocations, 45 (49%) did not report on subluxations, and 58 (64%) did not report on apprehension. Seventeen different definitions for recurrence of instability, 4 definitions of dislocations, and 8 definitions of subluxation were used.

**Conclusion:**

Recurrence rates are poorly specified and likely underreported in the literature, hampering comparison with results of other studies. This highlights the need for a consensus on definition of recurrence across shoulder instability studies. We recommend not using the definition recurrence of instability anymore. We endorse defining dislocations as a radiographically confirmed dislocation or a dislocation that is manually reduced, subluxations as the feeling of a dislocation that can be (spontaneously) reduced without the need for a radiographically confirmed dislocation, and a positive apprehension sign as fear of imminent dislocation when placing the arm in abduction and external rotation during physical examination. Reporting on the events resulting in a dislocation or subluxation aids in making an estimation of the severity of instability.

**Level of Evidence:**

Level IV, systematic review.

Depending on the risks for recurrent shoulder instability can be managed conservatively, with (arthroscopic) soft-tissue procedure, or (open) bony procedures.^1^ The arthroscopic Bankart repair is the most used procedure, including up to 87% of instability procedures.^2^ Several studies have assessed recurrence rates after shoulder instability surgery. The recurrence rate for the general population varies from 0% to 8% after Latarjet procedure^3^ to 3.4% to 35% after arthroscopic Bankart repair.^4^^,^^5^ Although most studies describe rates of recurrent dislocation (instability), there is no consensus on the definition of these terms. For example, Randelli et al.^5^ uses redislocation or subluxation as a definition of recurrent instability, whereas Gerometta et al ^4^ does not describe a definition of a recurrence of instability/dislocation. As a result, findings in previous studies were hard to compare.^6^ Kuhn^7^ has described that shoulder instability studies are procedure based and not condition based, resulting in unclear definitions of instability. He introduced the frequency, etiology, direction, and severity system for describing instability. Kennedy et al.^8^ has described that there is a wide variety of definitions of recurrence in the literature and that the recurrence rates vary according to level of evidence, age, follow-up time, and attrition rate. Although Kennedy et al.^8^ have noticed that there are many different definitions used in the literature, it is still unclear how many studies did not define these definitions. The purposes of our study were to determine the definitions for recurrence used in the literature, assess the consensus in using these definitions, and determine the impact of these definitions on recurrence rates. We hypothesized that for shoulder instability the definition of recurrence is poorly reported and that there is no consensus on the definition to be used.

## Methods

This is a systematic review of available literature on the definition of recurrent anterior shoulder instability and is performed according to the Preferred Reporting Items for Systematic Reviews and Meta-Analyses (PRISMA) guideline.^9^ No review protocol was identified for this study.

### Literature Search and Study Selection

A literature search was performed on August 5, 2020, in PubMed and EMBASE with predefined search terms (Appendix 1), including all studies mentioning recurrence rates in Dutch, German, Arabic, and English. The search was limited to studies between 2000 and 2020 to give insight into definition of recurrence in the most recent literature. The inclusion criteria included studies assessing recurrence rates after arthroscopic anterior shoulder instability surgery. Letters to the editors, instructional courses, animal/cadaver/in vitro studies, conference papers, and studies published in journals with an impact factor <1 at the time of the literature search were excluded. Studies wherein the definition of recurrence was not explicitly defined were excluded. First, the studies were selected on title and abstract using the predetermined inclusion and exclusion criteria by 2 authors (H.A. and J.W.C.) independently. Hereafter full-texts were screened and studies were cross-referenced to search for additional studies. Disagreement was resolved by discussion. Agreement between the 2 observers was assessed using Cohen’s kappa, which is a scale of agreement ranging from 0 to 1. A kappa 0.21 to 0.40 corresponds with fair agreement, 0.41 to 0.60 with moderate agreement, 0.61 to 0.80 with substantial agreement, 0.81 to 0.99 with near-perfect agreement, and 1.00 with perfect agreement.

### Data Extraction

First, we checked whether the authors reported on recurrence rates and how they defined recurrence of instability, subluxations, and dislocations. Afterward, the recurrence rates, dislocation rate, subluxation rate, and positive apprehension rate were extracted and presented. The methodological quality of each study was assessed separately by the same 2 authors using the Coleman Methodology Score.^10^ The total number of points correlates with poor (0-49 points), fair (50-69 points), good (70-84 points), or excellent (85-100 points) quality of the study.

## Results

### Study Selection

**Fig 1:**
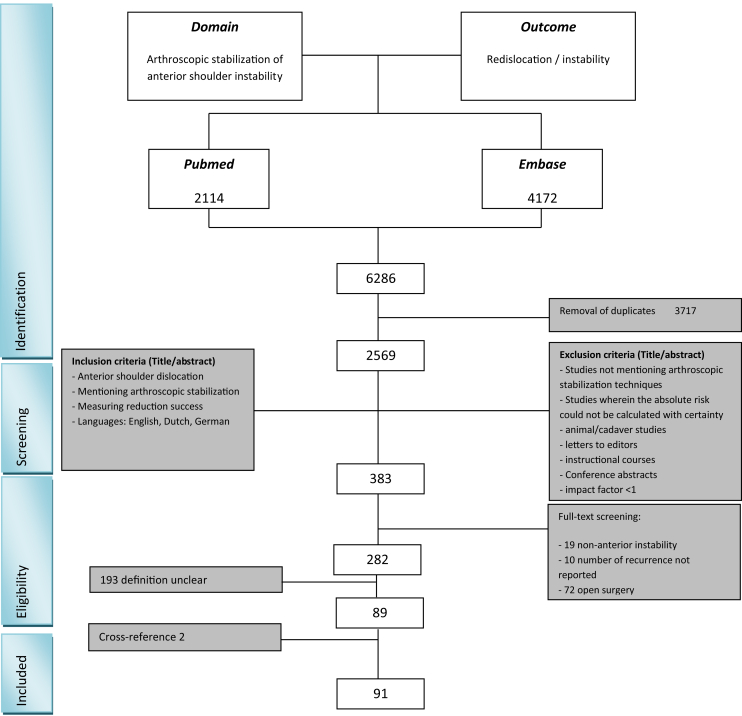
Flow chart. From 2569 studies in PubMed and EMBASE 282 are full-text screened, from which 89 studies are included.

In total, 2,569 titles and abstracts were screened, from which 383 studies were full-text screened resulting in 89 studies being included in the final analysis (Fig 1). From the 282 studies that were eligible for inclusion, 193 (68%) were excluded because the definition of recurrence was not defined clearly. Cross-referencing resulted in inclusion of 2 additional studies. The 2 observers agreed on 83.7% of the articles with a Cohen’s kappa of 0.67.

### Critical Appraisal and Definition of Recurrence

On the Coleman methodology score, one scored poor,^11^ 43 studies scored fair,^12^, ^13^, ^14^, ^15^, ^16^, ^17^, ^18^, ^19^, ^20^, ^21^, ^22^, ^23^, ^24^, ^25^, ^26^, ^27^, ^28^, ^29^, ^30^, ^31^, ^32^, ^33^, ^34^, ^35^, ^36^, ^37^, ^38^, ^39^, ^40^, ^41^, ^42^, ^43^, ^44^, ^45^, ^46^, ^47^, ^48^, ^49^, ^50^, ^51^, ^52^, ^53^, ^54^ 37 scored good,^57^, ^58^, ^59^, ^60^, ^61^, ^62^, ^63^, ^64^, ^65^, ^66^, ^67^, ^68^, ^69^, ^70^, ^71^, ^72^, ^73^, ^74^, ^75^, ^76^, ^77^, ^78^, ^79^, ^80^, ^81^, ^82^, ^83^, ^84^, ^85^, ^86^, ^87^, ^88^, ^89^, ^90^, ^91^ and 8 scored excellent^92^, ^93^, ^94^, ^95^, ^96^, ^97^, ^98^, ^99^ (Table 1). From the included studies only 30 studies (34%) reported on the definition of a subluxation, and 26 studies (29%) reported on the definition of a dislocation. In total 17 different definitions for recurrence were used, 8 definitions for subluxations were used, and 4 definitions of a dislocation (Tables 2 and 3). The most frequently used definitions for a recurrence were dislocation or a subluxation (DS) and dislocation, subluxation and/or apprehension (DSA). Fifty-two studies reported the definition of a recurrence as DS and 15 studies as DSA. The remaining 24 studies used 15 different definitions of a recurrence (Table 2).

**Table 1:** Coleman Analysis

Article (reference)	Number of Patients (n)	Part a						Part B			Mean Modified Coleman Score
		Mean Follow-up	Open/Arthroscopic	Diagnostic Certainty	Type of Study	Description of Treatments	Postoperative Rehabilitation	Outcome Criteria	Procedure for Assessing Outcomes	Description of Subjects	
Thal et al.^12^	72	24	+	+	Retrospective	+	+	1,3	2,3,4	1,2	69
Law et al.^13^	38	28	+	+	Retrospective	+	+	1,3	3,4	1,2	59
Wolf et al. ^53^	45	58	+	+	Retrospective	+	+	1,2,3	3,4	1	62
Park et al.^46^	20	29	+	+	Retrospective	+	+	1,2,3	3,4	1,2	61
Cho et al.^55^	72	25	+	+	Retrospective	+	+	1,2,3	3,4	1,2	69
Porcellini et al.^24^	385	36	+	+	Retrospective	+	+	1,2,3	—	1,2	61
Hantes et al.^93^	63	39	+	+	Prospective	+	+	1,2,3	1,2,3,4	1,2	94
Lützner et al.^35^	39	31	+/−	−	Retrospective	+	+	1,2,3	1,3,4	1	53
Flinkkilä et al.^58^	174	51	+	+	Retrospective	+	+	1,2,3	1,3,4	1,2	75
Imhoff et al.^69^	190	37	+	+	Retrospective	+	+	1,2,3,4	1,3,4	1	73
Park et al.^56^	161	37	+	+	Retrospective	+	+	1,2,3	2,3,4	1	69
Taverna et al.^14^	26	30	+	+	Retrospective	+	+	1,2,3	3,4	1	56
Kim et al.^80^	110	45	+	+	Retrospective	+	+	1,2,3	1,2,3,4	1,2	79
Van der Linde et al.^95^	70	108	+	+	Prospective	+	+	1,2,3,4	1,3,4	1,2	96
Gasparini et al.^91^	143	81	+	+	Retrospective	+	+	1,2,3	1,3,4	1	73
Kemp et al.^87^	40	24	+	−	Prospective	+/−	+	1,2,3	1,3,4	1,2	74
Ahmed et al.^88^	302	68	+	+	Retrospective	+	+	1,2,3,4	3,4	1	71
Kim et al.^52^	34	34	+	+	Retrospective	+	+	1,2,3	3,4	1	56
Sommaire et al.^86^	77	44	+	+	Retrospective	+	+	1,2,3	3,4	1,2	70
Milano et al.^94^	70	24	+	+	RCT	+	+	1,2,3,4	1,2,3,4	1	89
Owens et al.^57^	39	140	+	−	Prospective	+	+	1,2,3,4	1,3,4	—	75
Mohtadi et al.^89^	54	24	+/−	+	RCT	+	+	1,2,3	1,2,3,4	1,2	82
Shin et al.^90^	62	47	+	+	Retrospective	+	+	1,2,3	1,3,4	1	70
Tordjman et al.^54^	31	61	+	+	Retrospective	+	+	1,2,3	3,4	1,2	67
Robinson et al.^92^	84	24	+	+	RCT	+	+	1,2,3,4	1,2,3,4	1,2	94
Lee et al.^59^	170	38	+	+	Retrospective	+	+	1,2,3	3,4	1,2	70
Torrance et al.^15^	67	33	+	+	Retrospective	+	+	1,2,3	—	1,2	61
Vermeulen et al.^60^	147	76	+	+	Retrospective	+	+	1,2,3,4	1,3,4	1	76
Chan et al.^16^	131	24	+	+	Retrospective	+	+	1,2,3	—	1,2	61
Park et al.^61^	193	37	+	+	Retrospective	+	+	1,2,3	3,4	1,2	74
Ruiz Ibán et al.^17^	140	64	+	+	Retrospective	+/−	−	1,2,3	—	1	52
Su et al.^18^	65	56	+	+	Retrospective	+	+	1,2,3	—	1	59
Dickens et al.^19^	29	One season	+/−	−	Prospective	+/−	+	1,2,3	1	1	53
Chen et al.^20^	221	50	+	+	Retrospective	+	+	1,2,3	2	1	69
Moore et al.^21^	34	52	+	−	Retrospective	+	+	1,2,3	2,3,4	1	58
Yapp et al.^62^	33	170	+/−	−	RCT	+	+	1,2,3,4	1,2,3,4	1	81
Rhee et al.^63^	48	35	+	+	Retrospective	+	+	1,2,3	1,2,3,4	1,2	73
Oh et al.^64^	120	28	+	+	Retrospective	+	+	1,2,3	2,3,4	1,2	71
Ono et al.^65^	51	121	+	+	Retrospective	+	+	1,2,3	1,3,4	1	70
Nakagawa et al.^22^	140	24	+	+	Retrospective	+	+	1,2,3	1	1	61
Iizawa et al.^66^	68	31	+	+	Retrospective	+	+	1,2,3	1, 3,4	1,2	72
Lavoué et al.^23^	41	72	+/−	+	Retrospective	+	+	1,2,3	1,3,4	1	67
Pandey et al.^25^	136	49	+/−	+	Retrospective	+	+	1,2,3	3,4	1	62
Brzóska et al.^67^	100	83	+	+	Retrospective	+	+	1,2,3	1,2,3,4	1	77
Ernstbrunner et al.^26^	36	158	+/−	+	Retrospective	+	+	1,2,3	1,2,3,4	1	68
Gül et al.^27^	62	29	+	+	Retrospective	+	+	1,2,3	1,3,4	1	67
Loppini et al.^28^	670	101	+	+	Retrospective	+	+	1,2,3	—	1	62
Park et al.^29^	195	24	+	+	Retrospective	+	+	1,2,3	1,3,4	1	67
Jeon et al.^30^	118	28.2	+/−	+	Retrospective	+	+	1,2,3	3,4	1	59
O’Neill et al.^11^	20	24	+/−	+	Retrospective	+/−	−	1,2,3	1,3,4	1	48
Zimmermann et al.^31^	271	120	+/−	+	Retrospective	+	+	1,2,3	1,3,4	1	70
Flinkkilä et al.^32^	167	122	+	+	Retrospective	+	+	1,2,3	1,2,3,4	1	77
McRae et al.^96^	74	24	+	+	RCT	+	+	1,2,3	1,2,3,4	1	86
Bessière et al.^33^	93	72	+/−	−	Retrospective	+/−	+	1,2,3	1,2,3,4	1	64
Rose et al.^68^	65	63	+	+	Retrospective	+	+	1,2,3	1,3,4	1,2	78
Bessière et al.^34^	51	64	+/−	−	Retrospective	+/−	−	1,2,3,4	2,3,4	1,2	59
Castagna et al.^70^	65	63	+	−	Retrospective	+	+	1,2,3	1,3,4	1,2	73
Thomazeau et al.^36^	125	18	+	+	Prospective	+/−	+	1,2,3	1,3,4	1	62
Kim et al.^37^	59	77	+	−	Retrospective	+	+	1,2,3	3,4	1,2	66
Ozbaydar et al.^71^	93	47	+	+	Retrospective	+	+	1,2,3	1,3,4	1,2	75
Boileau et al.^72^	91	36	+	+	Retrospective	+	+	1,2,3	1,2,3,4	1	71
Calvo et al.^73^	61	45	+	+	Prospective	+	+	1,2,3	1,2,3,4	1,2	79
Kim et al.^97^	62	31	+	+	RCT	+	+	1,2,3	1,2,3,4	1,2	91
Kim et al.^74^	167	44	+	+	Prospective	+	+	1,2,3	1,3,4	1,2	75
Sperber et al.^38^	30	24	+/−	+	RCT	+/−	+	1,2,3	1,3,4	1	68
Nakagawa et al.^39^	257	24	+	+	Retrospective	+	+	1,2,3	—	1	56
Nakagawa et al.^40^	93	24	+	+	Retrospective	+	+	1,2,3	—	1,2	61
Chechik et al.^41^	83	46	+	−	Retrospective	+	+	1,2,3	3,4	1,2	65
Cole et al.^98^	37	36	+/−	+	RCT	+	+	1,2,3,4	1,2,3,4	1,2	85
Anderl et al.^42^	15	26	+	+	Prospective	+	+	1,2,3	1,3,4	1,2	62
Constantinou et al.^75^	32	217	+	+	RCT	+	−	1,2,3	1,2,3,4	1	79
De Giorgi et al.^43^	22	56	+	+	Retrospective	+	+	1,2,3	2,3,4	1,2	68
Salomonsson et al.^99^	62	120	+	−	RCT	+/-	+	1,2,3	1,2,3,4	1,2	87
Garcia et al.^44^	24	41	+	+	Retrospective	+/-	+	1,2,3,4	1,3,4	1,2	67
Armangil et al.^76^	72	49	+	+	Retrospective	+	+	1,2,3	1,3,4	1,2	75
Boughebri et al.^77^	45	79	+	+	Retrospective	+	+	1,2,3	1,3,4	1	70
Kim et al.^78^	36	42	+	+	Retrospective	+	+	1,2,3	1,2,3,4	1,2	73
McCabe et al.^45^	31	41	+	+	Retrospective	+	+	1,2,3	1,3,4	1	64
Ng and Kumar^79^	87	42	+	+	Prospective	+	+	1,2,3	1,2,3,4	1,2	79
Ee et al.^81^	79	24	+	+	Retrospective	+	+	1,2,3,4	1,3,4	1,2	75
Boileau et al.^47^	19	43	+	+	Retrospective	+	+	1,2,3,4	1,2,3,4	1	64
Sedeek et al.^48^	40	30	+	+	Retrospective	+	+	1,2,3,4	3,4	1,2	64
Phadnis et al.^49^	141	47	+	+	Case-control	+	+	1,2,3	—	1,2	64
Franceschi et al.^50^	50	25	+	+	Retrospective	+	+	1,2,3	2,3,4	1,2	68
Zhu et al.^82^	49	29	+	+	Retrospective	+	+	1,2,3	1,2,3,4	1,2	73
Mohtadi et al.^83^	83	24	+	−	RCT	+	+	1,2,3	1,2,3,4	1	81
Zaffagnini et al.^51^	49	164	+/−	−	Retrospective	+/-	+	1,2,3	1,3,4	1	57
Elmlund et al.^84^	76	98	+	−	Retrospective	+/-	+	1,2,3	1,2,3,4	1,2	72
Carreira et al.^85^	69	24	+	+	Retrospective	+	+	1,2,3,4	1,2,3,4	1	74
Gigis et al.^102^	38	36	+/−	+	Prospective	+	+	1,2,3	1,3,4	1	58
Shymon et al.^103^	71	29	+/−	−	Retrospective	+	−	1,2,3,4	1,3,4	1	57

**Table 2:** Results of the studies

Study	Patients Undergoing Arthroscopic Treatment	Definition of recurrence	Dislocation N (%)	Subluxation N (%)	Apprehension N (%)	Recurrence of Instability N (%)
Thal et al.^12^	72	DSA	4 (6)	1 (1)		5 (7)
Law et al.^13^	38	DSA	2 (5)		2 (5)	2 (5)
Wolf et al.^53^	45	DSA	2 (4)	0	5 (11)	7 (15.5)
Park et al.^46^	20	DSA	2 (10)	1 (5)		3 (16)
Cho et al.^55^	72	DSA	5 (7)		6 (8)	11 (15)
Porcellini et al.^24^	385	DS	31 (8)∗			31 (8)∗
Hantes et al. ^93^	63	DS	1 (2)	1 (2)	5 (8)	2 (3)
Lützner et al.^35^	39	DS	6 (15)	3 (8)	5 (14)	9 (23)
Flinkkilä et al.^58^	170	DS	15 (9)	18 (11)		33 (19)
Imhoff et al.^69^	190	DS	20 (11)	7 (4)		27 (14)
Park et al. ^56^	161	DS				12 (7)
Taverna et al.^14^	26	DS	0	0	1 (4)	0
Kim et al.^80^	110	DS	3 (3)	0	5 (5)	3 (3)
Van der Linde et al.^95^	70	DS	24 (35)			24 (35)
Gasparini et al.^91^	143	DS	19 (13)	14 (10)		33 (23)
Kemp et al.^87^	40	DS	2 (5)	6 (14)		8 (20)
Ahmed et al.^88^	302	DS	38 (13)	15 (5)		40 (13)
Kim et al.^52^	34	DS	2 (6)	0		2 (6)
Sommaire et al.^86^	77	DS requiring revision surgery	4 (5)	8 (10)		12 (16)
Milano et al.^94^	70	Dislocation	3 (4)			3 (4)
Owens et al.^57^	41	Dislocation requiring manual reduction, subluxation, or revision	6 (15)	9 (22)		15 (37)
Mohtadi et al.^89^	28	Self-report of 2 subluxation events or 1 dislocation	0	2 (7)		2 (7)
Shin et al.^90^	63	Dislocation or symptomatic instability	10 (16)	2 (3)		12 (19)
Tordjman et al.^54^	31	Walch-Duplay (< 51 points) + DS or Apprehension + feeling of instability	5 (16)	3 (10)		8 (26)
Robinson et al.^92^	42	Radiographic dislocation/subjective slipping or apprehension/Apprehension and load-and-shift test +	3 (7)			3 (7)
Lee et al.^59^	170	DS	12 (7)	20 (12)	20 (12)	32 (19)
Torrance et al.^15^	67	Dislocation or a subjective feeling of instability with objective clinical apprehension requiring further treatment				34 (51)
Vermeulen et al.^60^	147	DS	21 (14)	12 (8)		33 (22)
Chan et al.^16^	131	DS	22 (17)	12 (9)		34 (26)
Park et al.^61^	193	DS requiring revision surgery		6 (3)		13 (7)
Ruiz Ibán et al.^17^	140	DS			14 (10)	20 (14)
Su et al.^18^	65	DS				27 (42)
Dickens et al.^19^	29	DS				1 (3)
Chen et al.^20^	221	Dislocation or subluxation event that occurred within 2 years after surgery				31 (14)
Moore et al.^21^	34	DS	1 (3)	2 (6)		3 (9)
Yapp et al.^62^	32	DS	4 (12)	3 (9)		7 (21)
Rhee et al.^63^	48	DS requiring revision surgery	1 (2)	3 (6)		1 (2)
Oh et al.^64^	120	Dislocation or positive apprehension	12 (10)		14 (12)	26 (22)
Ono et al.^65^	51	DS	9 (18)		7 (14)	16 (31)
Nakagawa et al.^22^	140	DS				25 (18)
Iizawa et al.^66^	68	DS	17 (25) †			17 (25)†
Lavoué et al.^23^	41	DS	1 (2)	4 (10)	11 (27)	5 (1)
Pandey et al.^25^	136	DS	15 (11.0)			15 (11)
Brzóska et al.^67^	100	DSA				14 (14)
Ernstbrunner et al.^26^	36	Any redislocation requiring reduction by a third party or medical professional	6 (17)	3 (8)	3 (8)	6 (17)
Gül et al.^27^	62	Dislocation	5 (8)		8 (13)	5 (8)
Loppini et al.^28^	670	DS				114 (17)
Park et al.^29^	195	DS requiring revision surgery				15 (8)
Jeon et al.^30^	118	DSA				27 (23)
O’Neill et al.^11^	20	DSA				8 (40)
Zimmermann et al.^31^	271	DS	36 (13)	51 (19)	78 (29)	87 (32)
Flinkkilä et al.^32^	167	DS				50 (30)
McRae et al.^96^	74	At least one re-dislocation or minimum of 2 subluxations 6 weeks after operation				15 (20)
Bessière et al.^33^	93	DS	7 (8)	13 (14)	15 (16)	20 (22)
Rose et al.^68^	65	DS				14 (22)
Bessière et al.^34^	51	DS	6 (12)	6 (12)		12 (24)
Castagna et al.^70^	65	DS				14 (21)
Thomazeau et al.^36^	125	DS	2 (2)	2 (2)		4 (3)
Kim et al.^37^	59	DS			3 (5)	4 (7)
Ozbaydar et al.^71^	93	DS				10 (11)
Boileau et al.^72^	91	DS	6 (7)	8 (9)	9 (10)	14 (15)
Calvo et al.^73^	61	DS				11 (18)
Kim et al.^97^	62	DSA	0 (0)	0 (0)	4 (6)	4 (6)
Kim et al.^74^	167	DSA	1 (1)	2 (1)	4 (2)	7 (4)
Sperber et al.^38^	30	DS			0	7 (23)
Nakagawa et al.^39^	257	DS				42 (16)
Nakagawa et al.^40^	93	DS				22 (24)
Chechik et al.^41^	83	DS	9 (11)	7 (8)		16 (19)
Cole et al.^98^	37	DSA			3 (8)	9 (24)
Anderl et al.^42^	15	DSA	0 (0)	0 (0)	0 (0)	0 (0)
Constantinou et al.^75^	32	DS				6 (19)
De Giorgi et al.^43^	22	DS	4 (19)	1 (5)	3 (14)	5 (23)
Salomonsson et al.^99^	62	DS				34 (55)
Garcia et al.^44^	24	DS	4 (17)	6 (25)		10 (42)
Armangil et al.^76^	72	Dislocation	4 (6)		3 (4)	4 (6)
Boughebri et al.^77^	45	DS	4 (5)		4 (5)	4 (5)
Kim et al.^78^	36	DSA	1 (3)	2 (6)	1 (3)	4 (11)
McCabe et al.^45^	31	Dislocation, subluxation or revision instability surgery	1 (3)	3 (10)		11 (36)
Ng and Kumar^79^	87	DSA	2 (2)			2 (2)
Ee et al.^81^	79	Redislocation, any sensation of subluxation, or instability preventing return to full activity or requiring a further stabilizing procedure	6 (8)			6 (8)
Boileau et al.^47^	19	DS		1 (5)	2 (11)	1 (5)
Sedeek et al.^48^	40	Recurrent dislocation, symptomatic subluxation or instability preventing return to full active duties or necessitating an additional surgical stabilization procedure.				3 (8)
Phadnis et al.^49^	141	Recurrence of subluxation or frank dislocation or an ongoing or new feeling of instability	12 (9)			19 (13)
Franceschi et al.^50^	50	Subluxation, 1 or more frank dislocations, or at least 1 episode of dead arm syndrome	3 (6)	2 (4)	5 (10)	5 (10)
Zhu et al.^82^	49	DSA	1 (2)	2 (4)	1 (2)	4 (8)
Mohtadi et al.^83^	87	DS	16 (18)	4 (5)		20 (23)
Zaffagnini et al.^51^	49	redislocation	6 (12)			6 (12)
Elmlund et al.^84^	76	DS	8 (11)	6 (8)	6 (8)	14 (18)
Carreira et al.^85^	85	DS	4 (6)	3 (4)	2 (3)	7 (10)
Gigis et al.^102^	38	DS			4 (11)	5 (13)
Shymon et al.^103^	71	redislocation event and/or the need for further surgical intervention				17 (24)

∗ Only reported on dislocations.

† In a table the number is expressed as dislocations, while in the text as dislocations and subluxations.

**Table 3:** Definitions of Dislocation and Subluxation

Definition of dislocation
Dislocation needing reduction (by medical professional or third party)^12^^,^^16^^,^^19^^,^^26^^,^^31^^,^^33^^,^^34^^,^^37^^,^^57^^,^^58^^,^^68^^,^^70^^,^^72^, ^73^, ^74^^,^^87^^,^^91^^,^^102^
Objective documentation of a dislocation either radiologically or clinically^24^^,^^32^^,^^59^^,^^62^^,^^71^^,^^75^^,^^92^
Increased translation of the humerus relative to the glenoid to the point of complete separation of articular surfaces^96^
More than 1 episode of instability which needed manual reduction by other people^97^
Definition of subluxation
Instability without the need of reduction^12^^,^^13^^,^^16^^,^^26^^,^^31^^,^^33^^,^^34^^,^^45^^,^^57^^,^^58^^,^^68^^,^^70^^,^^72^^,^^87^^,^^91^^,^^97^^,^^102^
Subjective sense of subluxation/instability^24^^,^^37^^,^^44^^,^^60^^,^^69^^,^^71^^,^^99^
Sense of dislocation with a positive anterior apprehension test^59^
Transient instability event that did not require reduction but demonstrated a positive apprehension and relocation sign with radiographic or magnetic resonance imaging evidence of a Bankart or Hill-Sachs^19^
Symptomatic self-reported subluxation^62^
“Dead-arm” phenomenon or instability which spontaneously reduced^73^^,^^75^
Symptomatic translation of the humeral head relative to the glenoid articular surface without a dislocation^96^
Subluxation at the time of the clinical assessment or through a history of at least 1 episode of dead arm syndrome^84^

### Recurrence Rates Reported

Recurrence rates, as well as the rates of dislocation, subluxation and positive apprehension test results for each article, are reported in Table 2. In Park et al.,^56^ we could not extract the exact number of recurrent dislocations because only the amount of dislocations in group 1 were reported. Thirty studies (33%) did not report on recurrent dislocations, 45 studies (49%) did not report on recurrent subluxation, and 58 studies (64%) did not report on apprehension.

**Fig 2:**
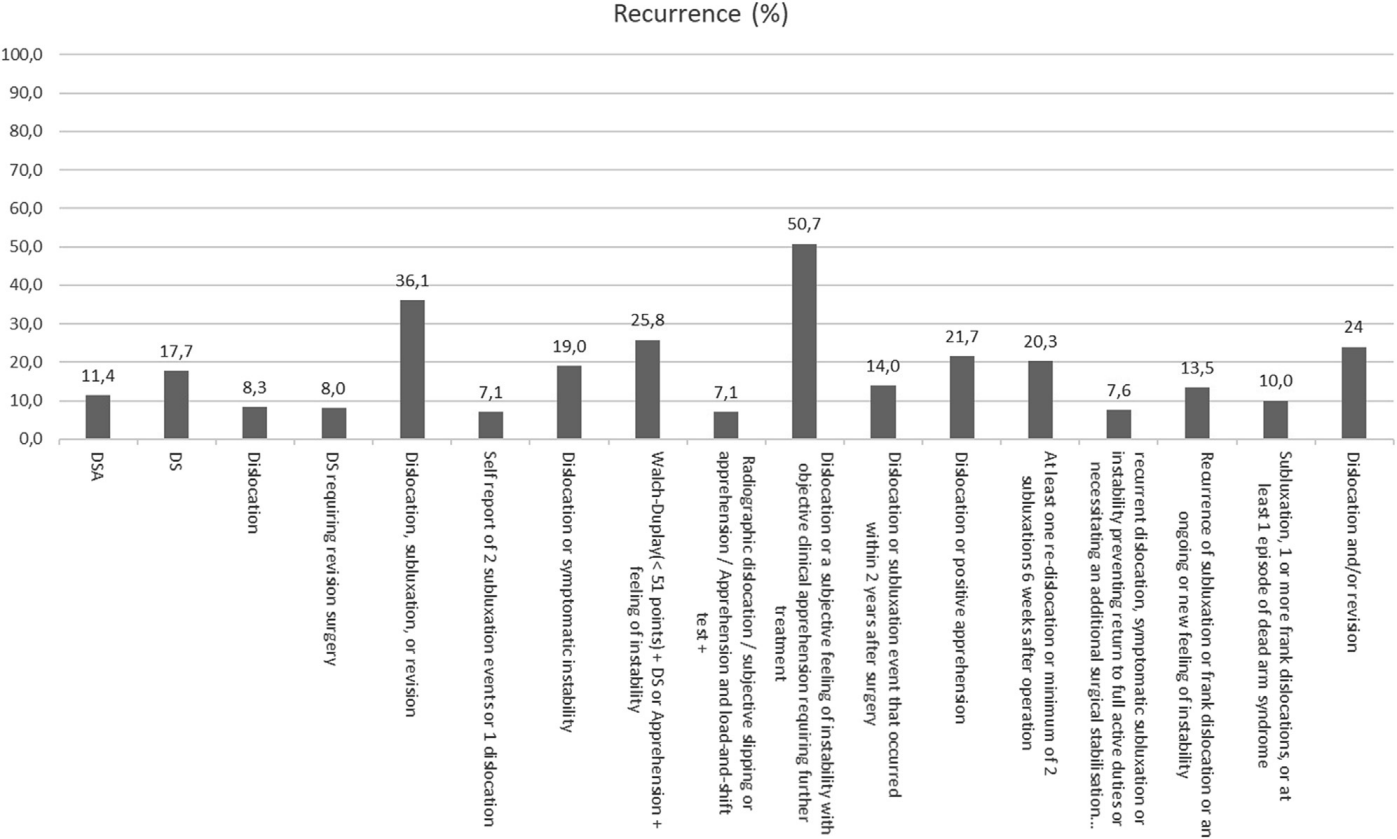
Recurrence percentage for each definition. This figure shows the percentage of recurrence for each definition of recurrence: on the X-axis we have the different definitions and on the Y-axis the percentage of patients who have suffered a recurrence. When no percentage is reported, it means that it has not been reported in any study.

**Fig 3:**
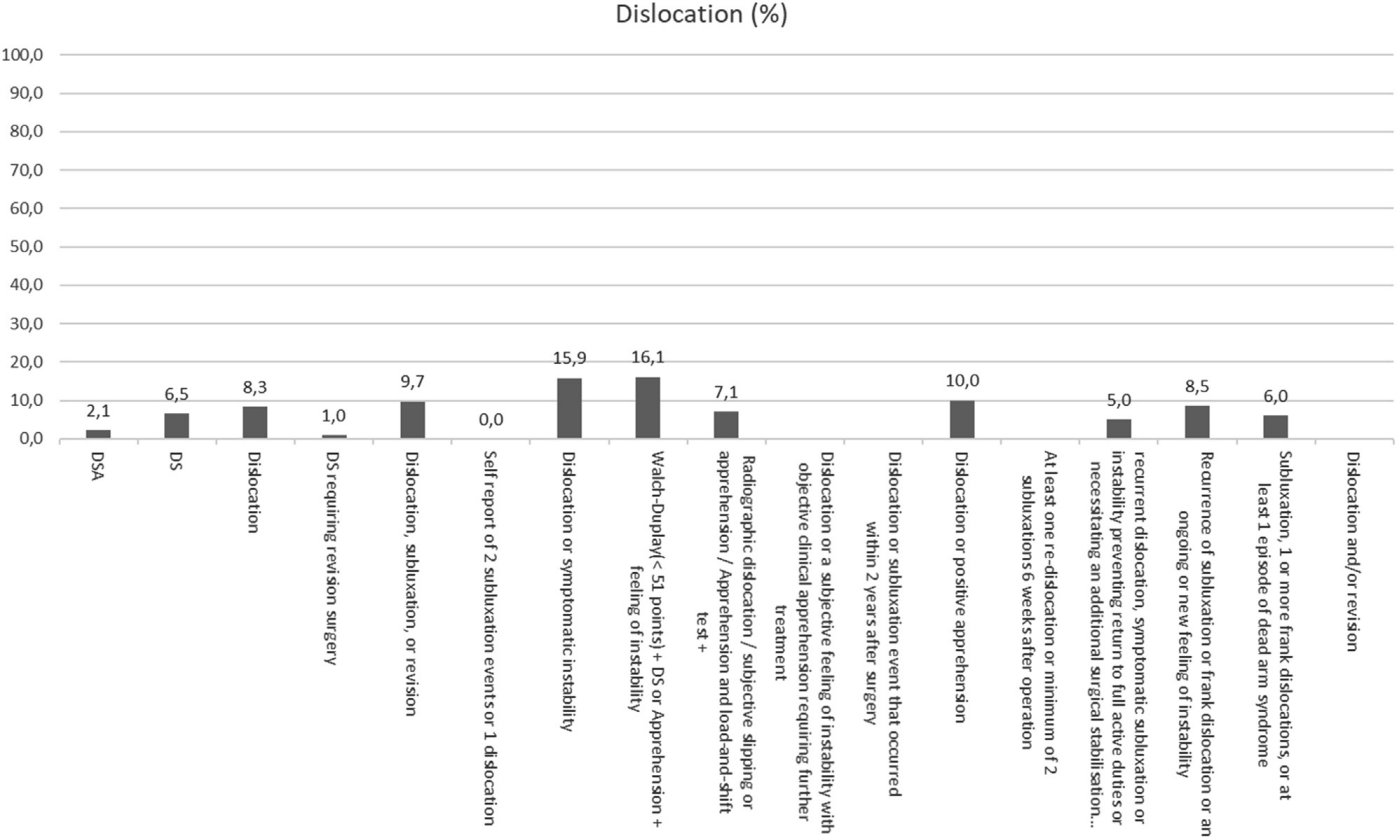
Dislocation percentage for each definition. This figure shows the percentage of dislocations for each definition of recurrence: on the X-axis we have the different definitions and on the Y-axis the percentage of patients who have suffered a dislocation. When no percentage is reported, it means that it has not been reported in any study.

**Fig 4:**
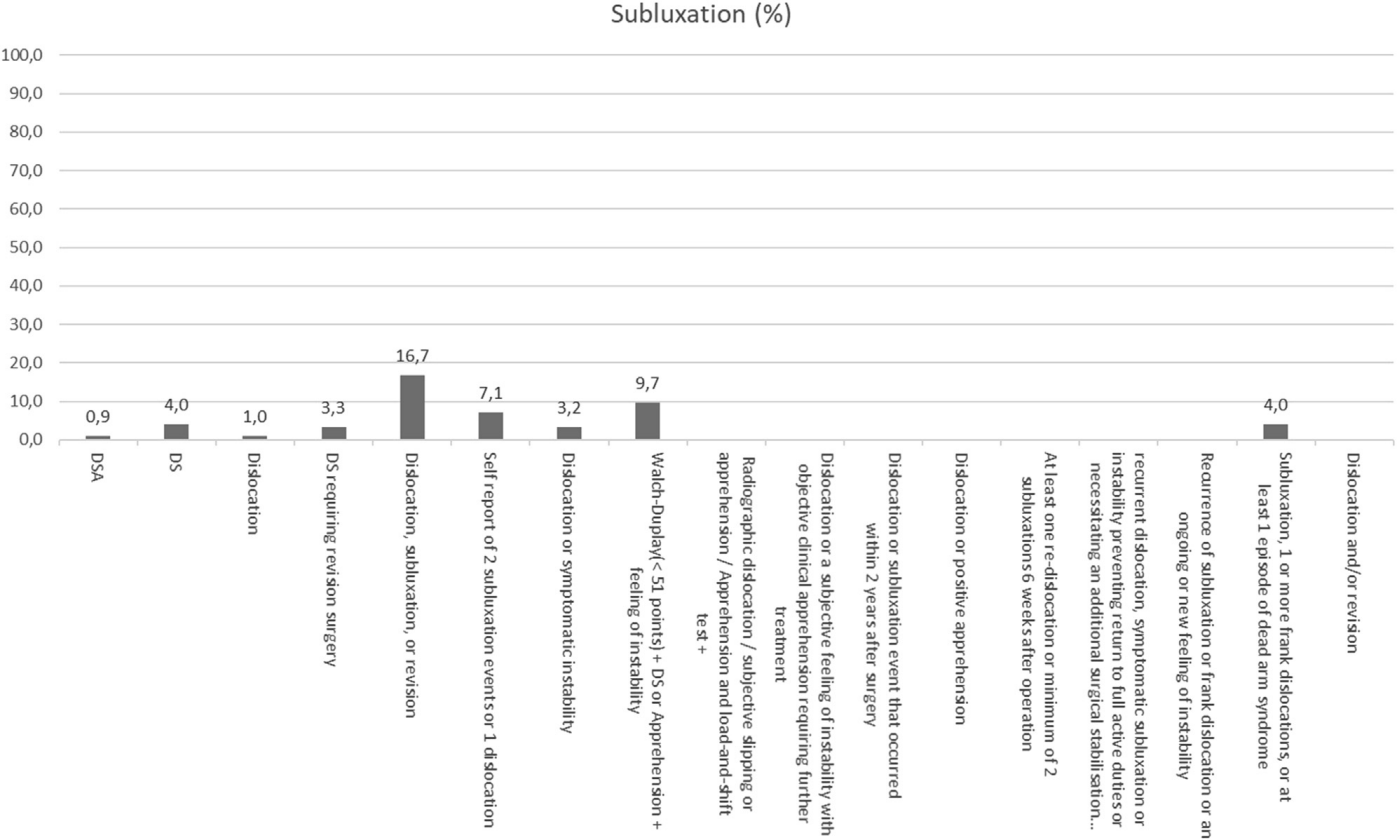
Subluxation percentage for each definition. This figure shows the percentage of subluxations for each definition of recurrence: on the X-axis we have the different definitions, and on the Y-axis the percentage of patients who have suffered a subluxation. When no percentage is reported, it means that it has not been reported in any study.

Overall recurrence rates ranged from 0% to 55%, dislocation rates from 0% to 35%, subluxation rates from 0% to 25%, and apprehension rates from 0% to 29%. The articles using the DSA definition had a total of 20 dislocations (2% from the studies reporting on dislocations), 8 subluxations (1%), 26 positive apprehension tests (3%) with an overall 107 recurrences (11%), whereas the studies defining recurrence as DS had 369 dislocations (7%), 225 subluxations (4%), 194 positive apprehension tests (3%), and an overall 1,006 recurrences (18%). Overall recurrence, dislocation, subluxation, and apprehension rates are shown in Fig 2, Fig 3, Fig 4 through 5. The articles varied in their reporting of dislocation, subluxation, and apprehension on the basis of the definitions of recurrence used (Table 4).

**Fig 5:**
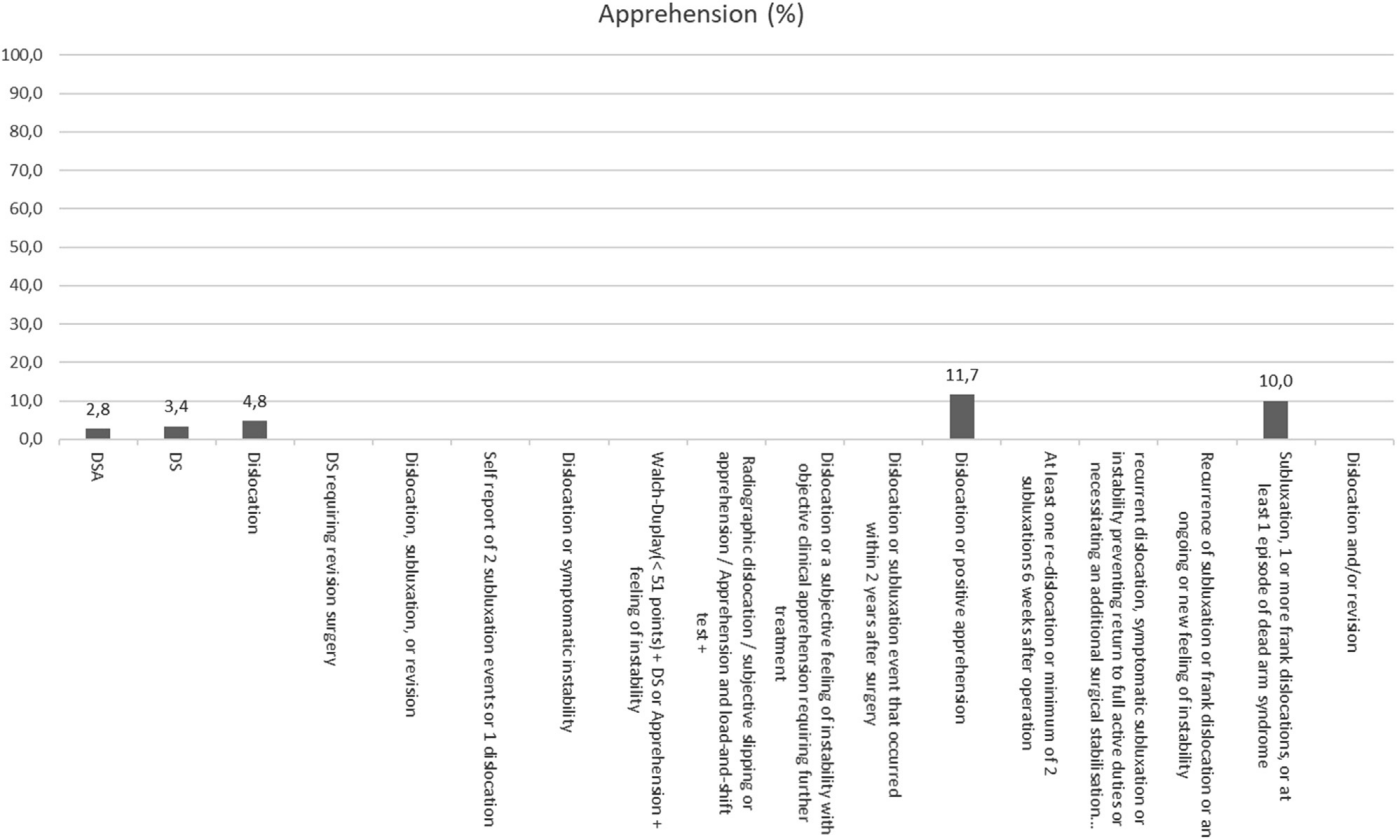
Apprehension percentage for each definition. This figure shows the percentage of apprehension for each definition of recurrence: on the X-axis we have the different definitions, and on the Y-axis the percentage of patients having a positive apprehension sign. When no percentage is reported, it means that it has not been reported in any study.

**Table 4:** Number of Dislocations, Subluxations and Recurrences Per Definition

Definition	Total	Dislocation N (%)	Subluxation N (%)	Apprehension (N)	Recurrence of Instability (N)
DSA	938	20 (2)	8 (1)	26 (3)	107 (11)
DS	5681	369 (7)	225 (4)	194 (3)	1,006 (18)
Dislocation	289	24 (8)	3 (1)	14 (5)	24 (8)
DS requiring revision surgery	513	5 (1)	17 (3)		41 (8)
Dislocation, subluxation, or revision	72	7 (10)	12 (17)		26 (36)
Self-report of 2 subluxation events or 1 dislocation	28	0 (0)	2 (7)		2 (7)
Dislocation or symptomatic instability	63	10 (16)	2 (3)		12 (19)
Walch-Duplay (<51 points) + DS or Apprehension + feeling of instability	31	5 (16)	3 (10)		8 (26)
Radiographic dislocation/subjective slipping or apprehension/Apprehension and load-and-shift test +	42	3 (7)			3 (7)
Dislocation or a subjective feeling of instability with objective clinical apprehension requiring further treatment	67				34 (51)
Dislocation or subluxation event that occurred within 2 years after surgery	221				31(14)
Dislocation or positive apprehension	120	12 (10)		14 (12)	26 (22)
At least one redislocation or minimum of 2 subluxations 6 weeks after operation	74				15 (20)
Recurrent dislocation, symptomatic subluxation or instability preventing return to full active duties or necessitating an additional surgical stabilization procedure.	119	6 (5)			9 (8)
Recurrence of subluxation or frank dislocation or an ongoing or new feeling of instability	141	12 (9)			19 (13)
Subluxation, 1 or more frank dislocations, or at least 1 episode of dead arm syndrome	50	3 (6)	2 (4)	5 (10)	5 (10)
Dislocation or revision	71				17 (24)

Percentages are percentages of total patients in studies mentioned to have an event (e.g., if a study does not report on subluxations, it is not used to calculate the percentage of subluxations)

## Discussion

The results of this study show that there are no uniform definitions of recurrence, subluxation, or dislocation after shoulder stabilization surgery used in the current literature. Using different definitions leads to a high level of heterogeneity. This could lead to misinterpretation of results and conclusions.

### Recommendations

To optimize readability and comparability of studies, we have made recommendations regarding the definitions of (recurrent) instability, dislocation, subluxation, and apprehension. For dislocations, we suggest the definition of a radiographically confirmed dislocation or a dislocation that is manually reduced. For this definition the shoulders reduced by a care giver or by patients themselves should be differentiated. To avoid underreporting of dislocations, all self-reported dislocation with signs of a sustained dislocation in further radiographs, such as Hill-Sachs or bony Bankart lesion in comparison with the preoperative situation, could be categorized as a confirmed dislocation. For subluxations, we advise using the definition of the feeling of a dislocation that can be (spontaneously) reduced without the need for a radiographically confirmed dislocation. For a positive apprehension sign, we suggest using the definition as mentioned by Lädermann et al.^100^ as fear of imminent dislocation when placing the arm in abduction and external rotation during physical examination. We suggest not using the definition recurrence of instability anymore to avoid using multiple meanings of this term; if used we suggest using the definition as a dislocation or a subluxation and also report on these events separately. We chose this definition because of the fact that dislocations and subluxations can be regarded as a (partial) failure of the operation, whereas a positive apprehension test result does not always correlate with instability of the shoulder. This is because a positive apprehension could be related to changes in functional cerebral networks induced by prior instability that can persist even after stabilizing the shoulder.^101^ Finally, we endorse reporting on the events resulting in a dislocation or subluxation to be able to make an estimation of the severity of instability. For example, a shoulder that dislocates during normal daily activities is potentially more unstable in comparison with a shoulder dislocating after a collision during sports.

### Limitations

Although DSA and DS have significantly different recurrence rates, the high number of studies not reporting dislocations, subluxations, and apprehension rates separately makes it unknown whether the recurrence rates would remain similar if all studies held the same criteria for defining recurrences in their cohort (e.g., not including apprehension in the definition could lead in less-reported recurrences). Because of corrections for multiple comparisons being not feasible for 17 definitions and because of the high variability in surgical techniques and patient characteristics, we did not compare the results for the different definitions. Remarkably, we had to exclude 68% of eligible studies because recurrence rates were not defined at all.

Another limitation of this study is that we could not compare the results of the different techniques to assess whether other definitions could lead to other results. We agree with the results Kuhn^7^ and Kennedy et al.^8^ The difference with Kennedy et al.^8^ is that we were stricter in whether a definition is explicitly defined to avoid overestimation of the reporting of recurrence rates; for example, in Kennedy et al.^8^ an article was regarded as defining recurrences as a dislocation if they only reported on dislocations without explicitly defining recurrences. Kasik and Saper^101^ have also reported that there are different definitions of recurrences after arthroscopic Bankart repair in the adolescent athletes. However, just like the article of Kennedy et al.,^8^ they also included articles that do not define recurrences explicitly.

## Conclusion

Recurrence rates are poorly specified and likely underreported in the literature, hampering comparison with results of other studies. This highlights the need for a consensus on definition of recurrence across shoulder instability studies. We recommend not using the definition recurrence of instability anymore. We endorse defining dislocations as a radiographically confirmed dislocation or a dislocation that is manually reduced, subluxations as the feeling of a dislocation that can be (spontaneously) reduced without the need for a radiographically confirmed dislocation, and a positive apprehension sign as fear of imminent dislocation when placing the arm in abduction and external rotation during physical examination. Reporting on the events resulting in a dislocation or subluxation aids in making an estimation of the severity of instability.

## **Appendix 1.** Search terms

### PubMed search:

((((Shoulder[Mesh] OR Shoulder Joint[Mesh] OR shoulder∗[tiab] OR glenohumeral[tiab]) AND (Joint Dislocations[Mesh] OR dislocation∗[tiab] OR ∗luxat∗[tiab] OR joint instability[Mesh] OR instability[tiab])) OR shoulder dislocation[Mesh]) AND (Recurrence[Mesh] OR recurren∗[tiab] OR relaps∗[tiab] OR redislocat∗[tiab] OR reluxat∗[tiab] OR resubluxat∗))

### EMBASE search:

(((shoulder∗ OR glenohumeral) AND (dislocation∗ OR ∗luxat∗ OR instability)) AND (recurren∗ OR relaps∗ OR redislocat∗ OR reluxat∗ OR resubluxat∗))

## Supplementary Data

ICMJE author disclosure forms
